# Anomalous Enhancement in MXene‐Based Electrochemical Actuators: Pseudo‐Capacitance‐Induced Multi‐Charge Accumulation in Confined Space

**DOI:** 10.1002/advs.77097

**Published:** 2026-08-14

**Authors:** Jiang Xu, Jipeng Feng, Bingqing Hu, Xude Li, Shifei Huang, Shanhai Ge, Guoxiu Wang

**Affiliations:** ^1^ College of Electrical Energy and Power Engineering Yangzhou University Yangzhou Jiangsu China; ^2^ Institute of Intelligent Flexible Mechatronics Jiangsu University Zhenjiang Jiangsu China; ^3^ School of Mechanical Engineering Yangzhou University Yangzhou Jiangsu China; ^4^ Department of Mechanical Engineering The Pennsylvania State University University Park Pennsylvania USA; ^5^ Centre for Clean Energy Technology School of Mathematical and Physical Sciences Faculty of Science University of Technology Sydney Sydney NSW Australia

**Keywords:** abnormal electrochemical behavior, charge storage mechanism, electrochemical actuator, in situ electrochemical characterization, titanium carbide MXene

## Abstract

Two‐dimensional (2D) transition‐metal carbides and nitrides (MXenes) exhibit high electronic conductivity, large volumetric capacitance, and excellent mechanical robustness, making them attractive for electrochemical actuators (ECAs). However, the fundamental link between surface chemistry, charge‐storage mechanism, and macroscopic actuation remains unclear, limiting their full potential. Here, we elucidate the actuation mechanism of Ti_3_C_2_‐based ECAs, revealing that actuation is driven by pseudocapacitive reactions rather than by simple ion intercalation. Controlled hydrothermal treatment of Ti_3_C_2_T*_x_* can tailor oxygen functional groups, boosting pseudocapacitive activity and interlayer adjustability. In situ structural analysis reveals that anomalous interlayer expansion arises from multiple hydrated ions accumulating at confined Ti─O─Ti active sites during pseudocapacitive reactions. Coupled with a beneficial wave‐like cross‐section morphology, the oxygen‐functionalized Ti_3_C_2_‐based solid‐state ECA achieves a peak‐to‐peak strain of ∼1% at 0.05 Hz under a ±1 V square‐wave voltage, which is four times greater than the pristine Ti_3_C_2_T*_x_* device, while maintaining excellent cycling stability. These findings culminate in a mechanistic framework linking atomic‐scale surface chemistry to macroscopic device performance, providing critical insights for designing advanced 2D material‐based actuators.

## Graphical abstract

We elucidate the electrochemical actuation mechanism of 2D material‐based electrodes within a MXene‐centered framework, linking atomic‐scale surface chemistry to macroscopic performance (see schematic). Mechanistically, the reduction of Ti─O─Ti sites in H_2_SO_4_ triggers the accumulation of hydrated ions at confined active sites, resulting in anomalous interlayer expansion.

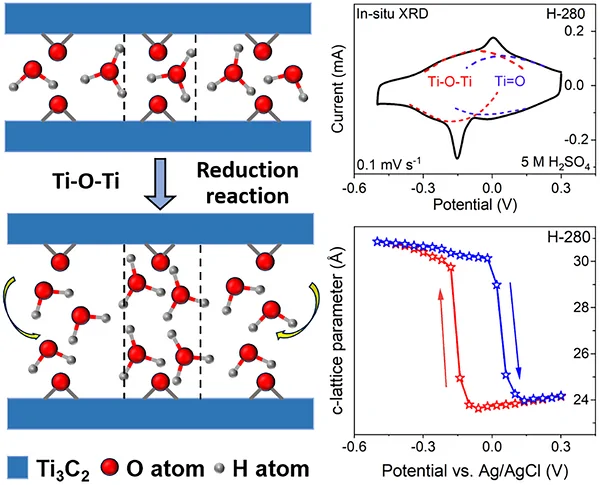

## 1 Introduction

Electrochemical capacitors, also known as supercapacitors, store charge through reversible electrochemical interactions at the electrode–electrolyte interface [1]. This core principle has been effectively adapted to directly convert electrical energy into mechanical work, leading to the development of electrochemical actuators (ECAs) [2]. Unlike conventional actuators, which rely on external stimuli such as pneumatic pressure, heat, or light [3, 4, 5, 6, 7, 8], ECA actuation primarily stems from reversible volume changes of electrodes, induced by ion adsorption and desorption during electrochemical processes. ECAs offer several distinct advantages, including high operational safety (due to low‐voltage driving and ambient‐temperature compatibility), lightweight, inherent softness, and precise controllability [2, 9, 10]. These attributes make ECAs a promising technology for applications in fields such as tactile displays, bionic devices, and soft robotic grippers [11].

A major breakthrough came in 1999 when Baughman et al. reported the first carbon nanotube (CNT)‐based actuators [2], which were driven by electrochemical double‐layer charging in aqueous electrolytes. Since then, various capacitive materials have been explored as electrodes for actuators, including conductive polymers [9, 12, 13], transition metal oxides [14, 15], and carbon nanomaterials [2, 16, 17, 18]. Nevertheless, achieving rapid actuation at low operating voltages (1 V) while maintaining high stability, mechanical strength, and flexibility remains a big challenge. Recently, 2D nanomaterials gained attention as promising ECA electrodes due to their high specific surface area, robust mechanical properties, and high electrical conductivity. Materials such as graphene [17, 18], carbon nitride [19], metallic MoS_2_ [20], and MXene [9, 11, 21] are at the forefront for ECA applications. Among these, Ti_3_C_2_T*_x_* MXene stands out, owing to its high volumetric capacitance [22, 23], excellent electronic/ionic conductivity [24, 25], and favorable polar surface for electrolyte wetting [23, 24, 26]. In addition, MXene exhibits a unique hybrid charge storage mechanism that combines surface redox pseudo‐capacitance with electrical double‐layer capacitor (EDLC)‐like intercalation capacitance [24, 27]. The tunability of its surface functional groups enhances pseudocapacitance behavior and promotes charge injection [24, 28, 29], which is expected to improve actuator performance. However, charge quantity alone does not fully determine actuation performance; charge distribution and microstructure of electrode materials also play critical roles. For instance, our previous study revealed that in carbon nanotubes relying solely on electric double‐layer adsorption, actuation performance varied considerably with charge–discharge rate due to electroosmotic effects, even when the corresponding capacitance changed only slightly [30]. Moreover, MXene‐based systems exhibit non‐classical structural evolution during charge storage. In situ X‐ray diffraction (XRD) analysis indicated that, under high‐potential operation in H_2_SO_4_ electrolyte, ion intercalation into MXene induced an unexpected contraction of its interlayer spacing [31], challenging the conventional assumption that ion insertion invariably leads to structural expansion. Thus, despite the great promise of MXene for high‐performance ECAs, this fundamental knowledge gap remains a major obstacle to their development.

Here, we synthesized Ti_3_C_2_ MXenes with tunable oxygen functional groups via hydrothermal nucleophilic substitution. These functional groups enhance the pseudocapacitive properties of the MXene. Using a combination of in situ electrochemical characterization and macroscopic electrochemical actuation measurements, we elucidated the actuation mechanism of 2D material‐based electrodes and established a MXene‐centered mechanistic framework linking atomic‐scale surface chemistry to macroscopic device performance. We propose a pseudocapacitive actuation mechanism in which the accumulation of multiple hydrated ions within confined interlayer spaces drives actuation, while preserving the structural integrity of the MXene.

## 2 Results and Discussion

### 2.1 Structures of Pristine Ti_3_C_2_T*_x_* and Oxygen‐Functionalized Ti_3_C_2_ MXene Films

Most ECA electrodes have a bilayer structure for bending and force generation, relying on the in‐plane strain difference between the active material layer and the substrate, as well as the electrode modulus. As shown in Scheme 1a (α↔α’), an orderly arrangement of 2D materials induces only axial deformation and thus cannot produce actuation. True actuation arises from two essential structural characteristics: anisotropic organization of the 2D materials and interlayer heterogeneity. Together, these enable the in‐plane deformation of the active material layer (Scheme 1a, β↔β’, γ↔γ’).

**SCHEME 1:**
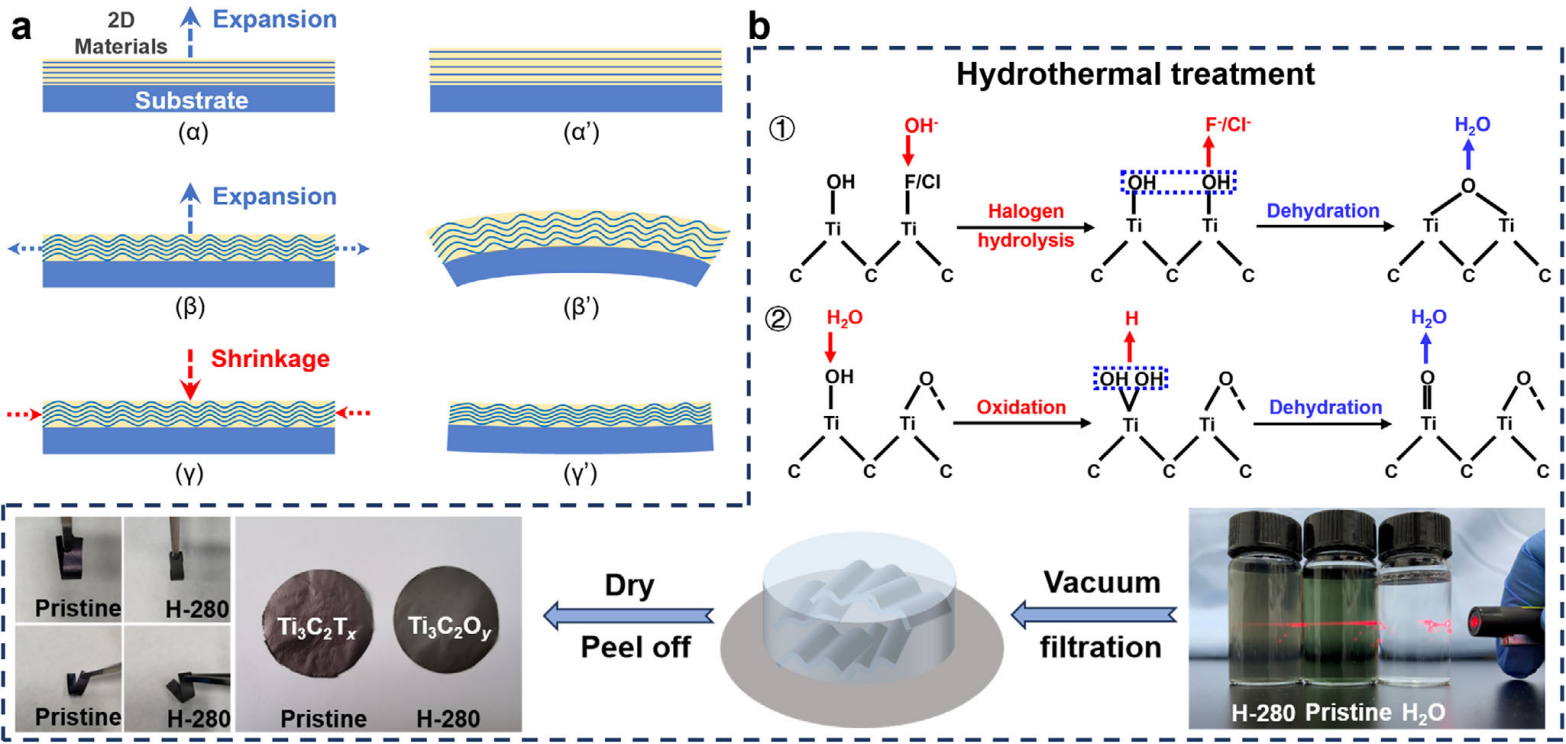
(a) Schematic illustrating the working principle of a 2D material‐based electrochemical actuator. (b) Schematic of the preparation process for high‐density oxygen‐terminated Ti_3_C_2_ MXene.

During MXene colloidal solution preparation, sonication‐assisted exfoliation yields single‐ or few‐layer nanosheets with lateral dimensions of ∼100 nm and heterogeneous shapes (Figure 1a). The irregular stacking of these nanosheets during vacuum filtration results in an anisotropic internal structure within the MXene film, as confirmed by the cross‐sectional SEM and TEM images of the film (Figure 1c,e,g). Moreover, a notable wave‐like morphology has been observed, which further facilitates in‐plane deformation (Figure S1). Consequently, interlayer variation emerges as a critical parameter governing actuation in MXene‐based actuators.

**FIGURE 1:**
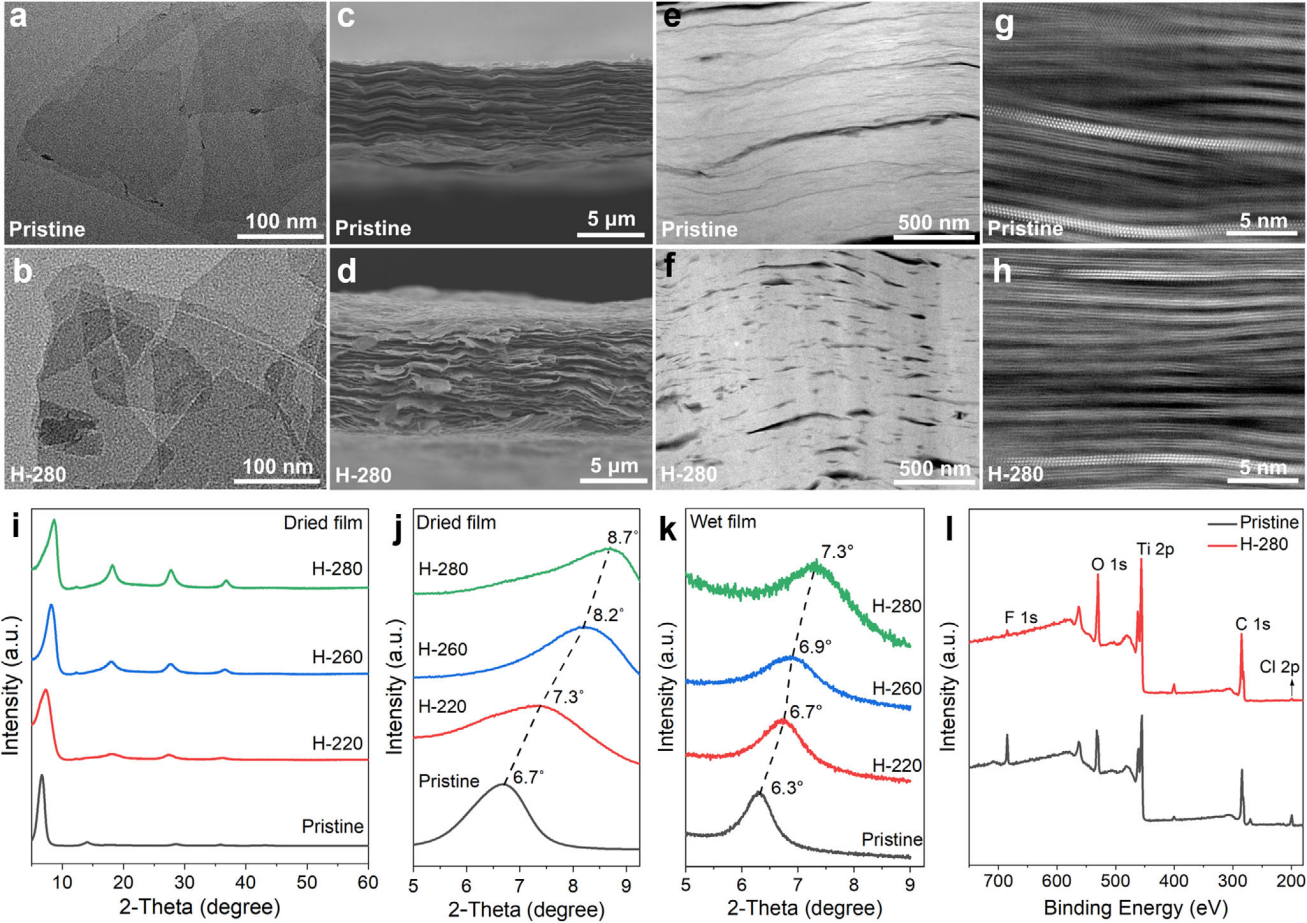
**C**omparative characterization of pristine Ti_3_C_2_T*_x_* and oxygen‐functionalized Ti_3_C_2_ MXene. TEM images of (a) pristine and (b) H‐280 samples. Cross‐sectional FE‐SEM images of (c) pristine and (d) H‐280 vacuum‐filtered films. Cross‐sectional HAADF‐STEM images of (e, g) pristine and (f, h) H‐280 films. (i) XRD patterns of pristine, H‐220, H‐260, and H‐280 films. Enlarged view of the (002) peak in XRD patterns of MXene films (j) before and (k) after immersion in 5 M H_2_SO_4_. (l) XPS spectra of the pristine Ti_3_C_2_T*_x_* and H‐280 films.

Given that MXene exhibits both pseudocapacitive and intercalation capacitance behaviors in acidic electrolytes, we sought to clarify how charge storage affects its interlayer spacing. To this end, a series of MXenes derived from pristine Ti_3_C_2_T*_x_* were synthesized with controlled ratios of oxygen‐containing functional groups via an alkaline hydrothermal nucleophilic reaction (Scheme 1b, Figures S2 and S3, hydrothermally treated samples at different temperatures are denoted as H‐T, where T is the hydrothermal temperature in °C). As shown in Figure 1b,h, the layered Ti_3_C_2_ structure is well maintained even after hydrothermal treatment at 280°C for 20 h, as well as the wave‐like structure in the film (Figure 1d,f). This structural stability is further confirmed by XRD patterns (Figure 1i). To further verify that hydrothermal treatment replaces halogen terminals (─F/─Cl) on Ti_3_C_2_T*_x_* with oxygen‐containing functional groups, XPS measurements were performed to monitor the evolution of surface chemistry. Consistent with the EDS results (Figure S3), only trace amounts of halogens were detected in the XPS spectrum of H‐280 (Figure 1l). Furthermore, the pronounced reduction in hydroxyl groups (Figure S4), coupled with the intact Ti_3_C_2_ framework, suggests that the surface terminations are predominantly transformed into oxygen‐containing species.

Furthermore, with increasing hydrothermal temperature, the (002) diffraction peak of the dried films gradually shifts to higher angles, reflecting the replacement of hydroxyl and halogen terminal groups with oxygen‐containing groups and the corresponding reduction in interlayer spacing [24, 32]. Upon immersion in electrolyte, all films exhibit an expansion in interlayer spacing. Notably, the hydrothermally treated samples show a substantially larger increase than the pristine film (Figure 1j,k). For the H‐280 film, severe shrinkage during drying induces interlayer tearing (Figure S1), as unambiguously revealed by cross‐sectional TEM imaging (Figure 1f). We therefore hypothesize that the H‐280 film will exhibit limited macroscopic actuation upon electrolyte immersion, as the expansion primarily compensates for the interlayer tears generated during drying, thereby suppressing large‐scale dimensional changes. This hypothesis is confirmed by the actuation tests presented in the following section.

### 2.2 Electrochemical Actuation Performance of MXene Film in Acidic Electrolyte

The actuation performance of MXene‐based films was characterized using a bilayer configuration (MXene|Kapton tape) in a three‐electrode electrochemical system with 5 M H_2_SO_4_ as the electrolyte (Figure 2a). After three initial conditioning cycles, both the pristine and H‐280 films exhibited stable and reproducible cyclic voltammetry (CV) at 5 mV s^−^^1^, indicating good electrochemical stability. Electrochemical analysis reveals that the pristine Ti_3_C_2_T*_x_* electrode exhibits composite capacitive behavior. A quasi‐rectangular region at high potential corresponds to intercalation capacitance with an EDLC character, while a pair of well‐defined peaks is associated with faradaic pseudocapacitance (Figure 2b). The latter is attributed to the redox reaction of Ti─O─Ti bonds with the MXene structure [33]. In contrast, the H‐280 surface is enriched with oxygen‐containing functional groups (including Ti─O─Ti and Ti═O), resulting in two well‐defined redox couples in the CV curves. The oxygen‐functionalized MXene delivers an ultrahigh specific capacitance of 821.8 F g^−^^1^ within the voltage window of −0.5 to 0.3 V, representing a nearly fourfold increase relative to the pristine sample (216.9 F g^−^^1^) and approaching the theoretical capacitance limit [24] of MXene materials (Figure 2c).

**FIGURE 2:**
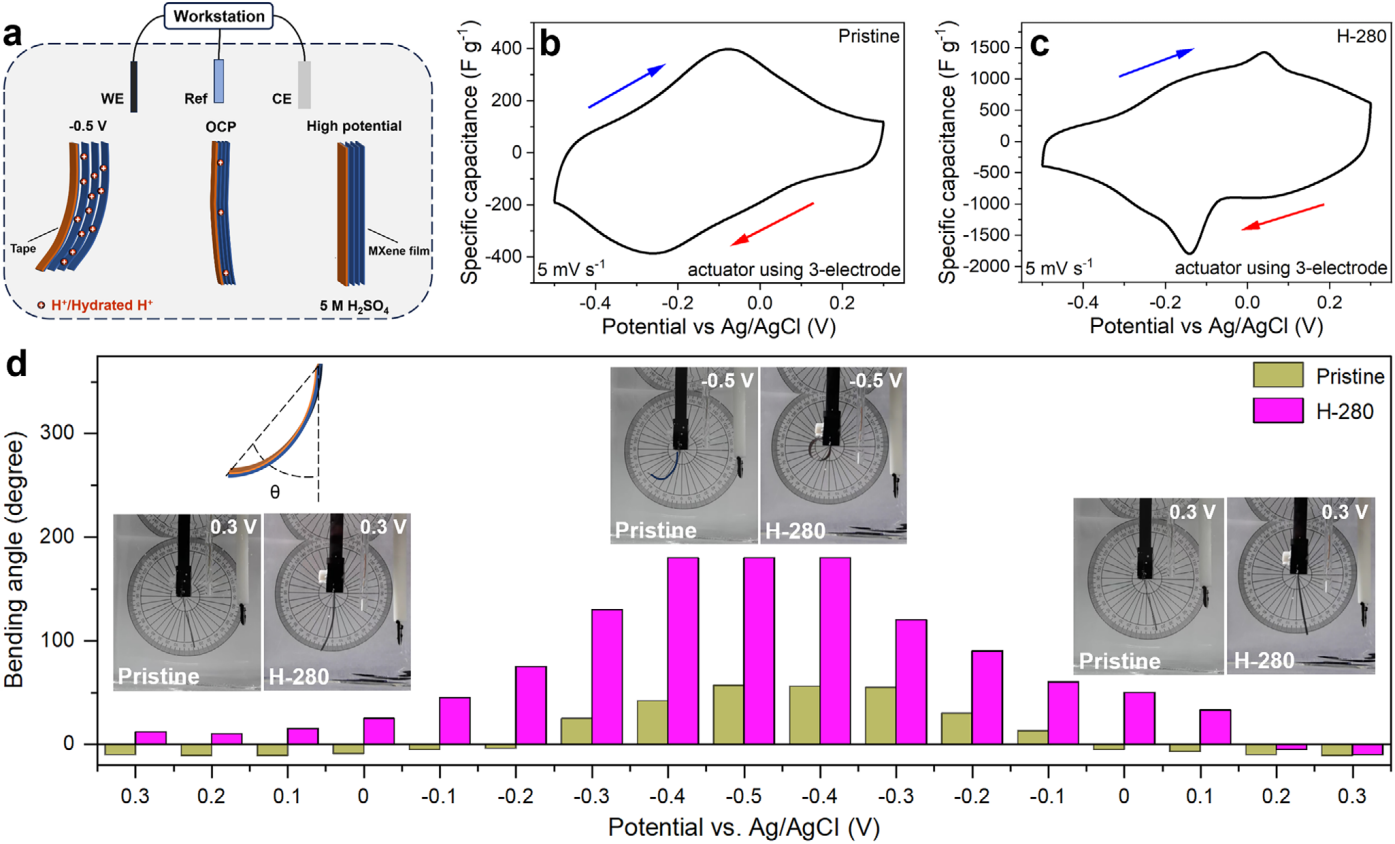
Electrochemical actuation performance of MXene in 5 M H_2_SO_4_ electrolyte. (a) Schematic illustration of a MXene‐based actuator operating in a three‐electrode system with 5 M H_2_SO_4_. CV curves of actuators based on (b) pristine and (c) H‐280 MXene, measured at a scan rate of 5 mV s^−1^ in 5 M H_2_SO_4_. (d) Bending angles of actuators based on pristine and H‐280 MXene under various applied potentials. The insets in (d) show optical images of the actuator electrodes at corresponding potentials.

The actuation performance of the MXene film electrodes was evaluated by measuring their bending angles (Figure S5, Videos S1 and S2). As depicted in Figure 2d, the pristine sample exhibits negligible deformation in the high‐potential region (0.3↔0 V), where charge storage is dominated by EDLC‐type capacitance. Noticeable actuation emerges only upon the onset of pseudocapacitive reactions (at ∼0 V). However, further charge injections from −0.4 to −0.5 V do not produce a proportional increase in bending angle. In contrast, the H‐280 sample demonstrates substantially enhanced actuation, rapidly achieving a 180° bend. Its maximum bending angle could not be quantified due to obstruction by the graphite electrode clamps. Nonetheless, in the high‐potential region (0.3–0 V), even substantial pseudocapacitive charge injection, it does not induce significant deformation. These observations indicate that the deformation of the MXene‐based actuator is associated with pseudocapacitance processes but is not directly proportional to the charge. Although the MXene film exhibits complex actuation behavior in acidic electrolyte, the deformation remains reversible and consistent with the CV response. A mechanistic understanding of this behavior is therefore critical for improving actuation performance and enabling precise control of MXene‐based actuators.

### 2.3 Actuation Mechanisms of MXene Film in Acidic Electrolyte

To elucidate the actuation mechanisms of MXene, we employed multiple in situ characterization techniques, including XRD, ultraviolet‐visible spectroscopy (UV–vis), and electrochemical quartz microbalance (EQCM), to probe its microstructural evolution, valence‐state changes, and mass variations during charge injection, respectively. In situ XRD performed in a modified three‐electrode cell (see Experimental Section) reveals the atomic‐scale structural evolution of MXene films with varying oxygen‐termination densities (Figure 3a–h). During CV cycling, the (002) diffraction peak of all samples shifted to varying degrees while exhibiting a consistent overall trend. In the high‐potential range (0.3 to −0.1 V) during discharge, the interlayer spacing of all samples gradually decreases with charge injection. For the pristine sample, this contraction is attributed to electrostatic screening, whereby the injected charges offset the intrinsic interlayer repulsion between negatively charged MXene sheets [31]. In contrast, for the hydrothermally treated samples, although substantial Ti═O pseudocapacitive reactions occur within this potential range, interlayer contraction still persists. Moreover, for H‐280, the terminal groups are mainly Ti─O─Ti and Ti═O species. Upon immersion in the electrolyte, a portion of the Ti═O active sites undergo pseudocapacitive protonation, where H_3_O^+^ ions are strongly adsorbed by Ti═O sites, resulting in an open‐circuit potential of 0.18 V (Figure S6). At 0.3 V, although some hydrated ions (H_3_O^+^) desorb from the Ti═O sites, a fraction remains intercalated, maintaining an expanded interlayer spacing compared with the dry film; the (002) peak position of the H‐280 electrode (2*θ* = 7.3°) coincides with that of the pre‐soaked sample (Figures 1k, and 3d). Concurrently, the film shows minimal curling, and its curvature remains comparable to that in the dried state (inset in Figure 2d and Figure S1). These observations suggest that the enlarged interlayer spacing during pre‐soaking effectively compensates for the pores generated by tearing during film drying (Figure 1f), thereby supporting the structural hypothesis proposed above.

**FIGURE 3:**
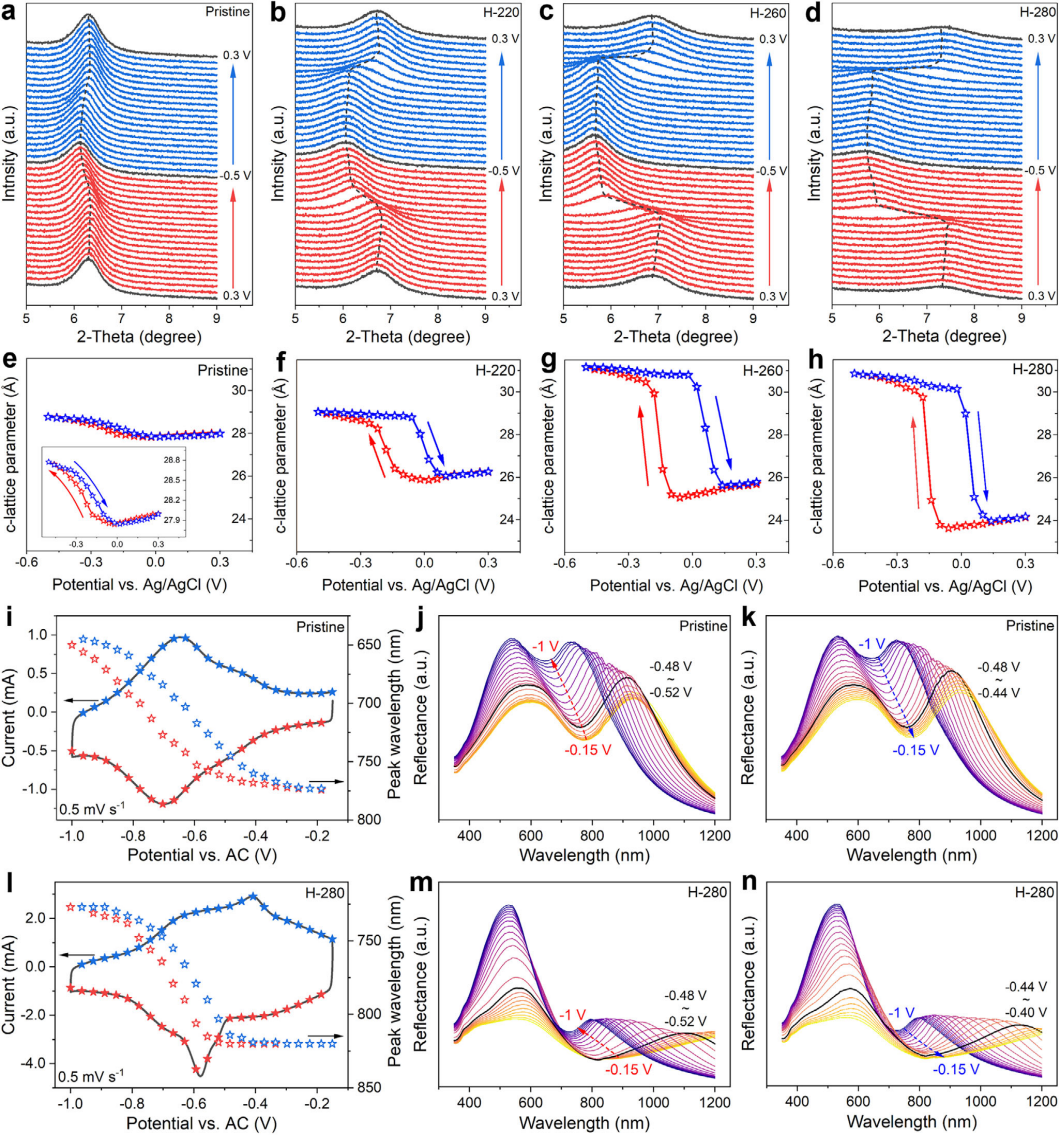
In situ XRD and UV–vis spectra analysis of MXene in 5 M H_2_SO_4_ electrolyte. In situ XRD patterns collected during CV cycling at 0.1 mV s^−1^ for film electrodes of (a) pristine, (b) H‐220, (c) H‐260, and (d) H‐280. Evolution of the *c*‐lattice parameter as a function of applied potential for (e) pristine, (f) H‐220, (g) H‐260, and (h) H‐280. CV curves of (i) pristine and (l) H‐280 MXene *vs*. activated carbon (AC), measured in 5 M H_2_SO_4_ at a scan rate of 0.5 mV s^−1^. In situ UV–vis spectra of (j, k) pristine and (m, n) H‐280 measured at selected potentials during charge and discharge.

As discharge proceeds, the interlayer spacing of all MXene samples increases sharply beginning at ‐0.1 V, with the expansion rate increasing with oxygen‐termination density. For example, H‐280 exhibits a 25% increase in its c‐lattice parameter (from 24 to 30 Å) within a narrow potential window of ∼0.05 V. Further charge injection, however, leads to merely marginal additional expansion. Notably, replacing ─OH, ─F, and ─Cl functional groups with ─O─ and ═O groups progressively reduce the initial interlayer spacing (from 28 to 24 Å), thereby amplifying the overall extent of spacing variation during cycling. Despite this, a clear threshold emerges in the maximum attainable interlayer spacing. Specifically, the pristine and H‐220 samples reach comparable maximum values (∼29 Å, *c*‐lattice parameter), whereas H‐260 and H‐280 achieve larger spacing (∼32 Å). Although no direct correlation is observed between the maximum interlayer spacing and the total measured capacitance (Figure S7), a clear relationship with the pseudocapacitive contribution is evident. These results indicate that structural expansion is governed primarily by Faradaic charge‐storage processes rather than by the overall capacitive output.

To probe the electrochemical driving mechanism of MXene film electrodes, we performed in situ UV–vis spectroscopy. Owing to its high sensitivity to valence‐state changes of Ti─O/═O species during pseudocapacitive reactions [24, 27] measurements were conducted in a custom‐built three‐electrode cell, with the MXene film serving as the working electrode in reflectance mode (Figure S8). As shown in Figure 3i–k, within the high‐potential range of −0.4 – −0.15 V vs. AC—corresponding to the quasi‐rectangular EDLC‐dominated region—the absorption peak remains nearly unchanged at ∼780 nm. Upon further decreasing the potential, the peak undergoes a significant blue shift, ultimately stabilizing at ∼640 nm. The closed‐loop evolution of the peak position indicates highly reversible valence‐state changes in pristine Ti_3_C_2_T*_x_* during cycling, whereas the observed hysteresis suggests kinetic limitations within this potential window. In contrast, in H‐280 (Ti_3_C_2_O*_y_*), where oxygen terminations dominate, nearly all surface titanium species are spectroscopically active in the UV–vis spectrum. The presence of high‐valence Ti═O groups results in a notable red shift (∼820 nm) in the initial spectrum relative to the pristine Ti_3_C_2_T*_x_* (Figure 3l–n). Electrochemically, H‐280 exhibits behavior similar to that of pristine Ti_3_C_2_T*_x_*, with minimal spectral shift in the high‐potential range (−0.4–−0.15 V). Upon further decreasing the potential, the peak undergoes a pronounced blue shift, ultimately stabilizing at ∼700 nm—slightly higher than that observed for Ti_3_C_2_T*_x_*, where the response is primarily associated with Ti─O─Ti species. This difference likely arises from the single‐electron transfer process of Ti═O groups. The comparable magnitude of wavelength shift indicates that Ti─O/Ti═O species in H‐280 actively participate in the redox process within this potential window, suggesting that charge transfer involves nearly all surface Ti atoms of Ti_3_C_2_. This collective Faradaic contribution underpins the exceptionally high capacity of H‐280.

Notably, after synchronizing the potential axes of the in situ XRD and UV–vis CV data (Figure S9), the evolution of interlayer spacing in both pristine Ti_3_C_2_T*_x_* and H‐280 MXene films closely mirrors the variation in the UV–vis spectral peak position. This strong correspondence indicates that the interlayer spacing variation during cycling in acidic electrolytes is governed by the pseudocapacitive reactions of Ti─O─Ti species. However, analysis of the entire Ti─O─Ti reaction process reveals that the structural evolution cannot be attributed solely to simple ion injection. Instead, it involves more complex electrochemical–structural coupling, as evidenced by the negligible change in interlayer spacing during charge injection within the high‐potential region.

To further elucidate this mechanism, EQCM measurements were conducted on MXene‐coated gold quartz crystals in H_2_SO_4_ electrolyte to investigate charge storage and ion transport processes and clarify the electrochemical actuation behavior of MXene electrodes. This technique enables *operando* gravimetric tracking in the working electrode, providing valuable insights into ion fluxes during electrochemical polarization [34]. A potential window of −0.25 to 0.25 V vs. Ag/AgCl was selected to encompass the pseudocapacitive reaction region while avoiding hydrogen evolution. Additionally, a narrower window (0–0.25 V) was applied to isolate ion transport behavior in the EDLC‐like intercalation regime. As shown in Figure 4a,c, the CV profile and corresponding mass response of Ti_3_C_2_T*_x_* at 5 mV s^−1^ exhibit overlapping start and end points in the mass loop, indicating stable and reversible charge transfer within the selected potential range. Within the EDLC‐dominated intercalation region (0–0.25 V vs. Ag/AgCl), the mass change scales almost linearly with accumulated charge (Figure 4b). According to Faraday's law, the average molecular weight per transferred charge is calculated to be 5.8 g mol^−1^, approximately one‐third of that of H_3_O^+^ (19 g mol^−1^). This result indicates the co‐transport of protons (H^+^) and hydronium ions during electrochemical cycling (Figure 4g). Although H^+^ is the smallest and lightest among all cations [35, 36], it seldom acts as an independent charge carrier because of its strong tendency to associate with water and form H_3_O^+^, a process accompanied by a high dehydration energy (11.66 eV for H_3_O^+^ → H^+^ + H_2_O) [37]. In other words, H_3_O^+^ is typically the charge carrier rather than H^+^ unless a continuous hydrogen‐bond network enables proton hopping via the Grotthuss mechanism [38].

**FIGURE 4:**
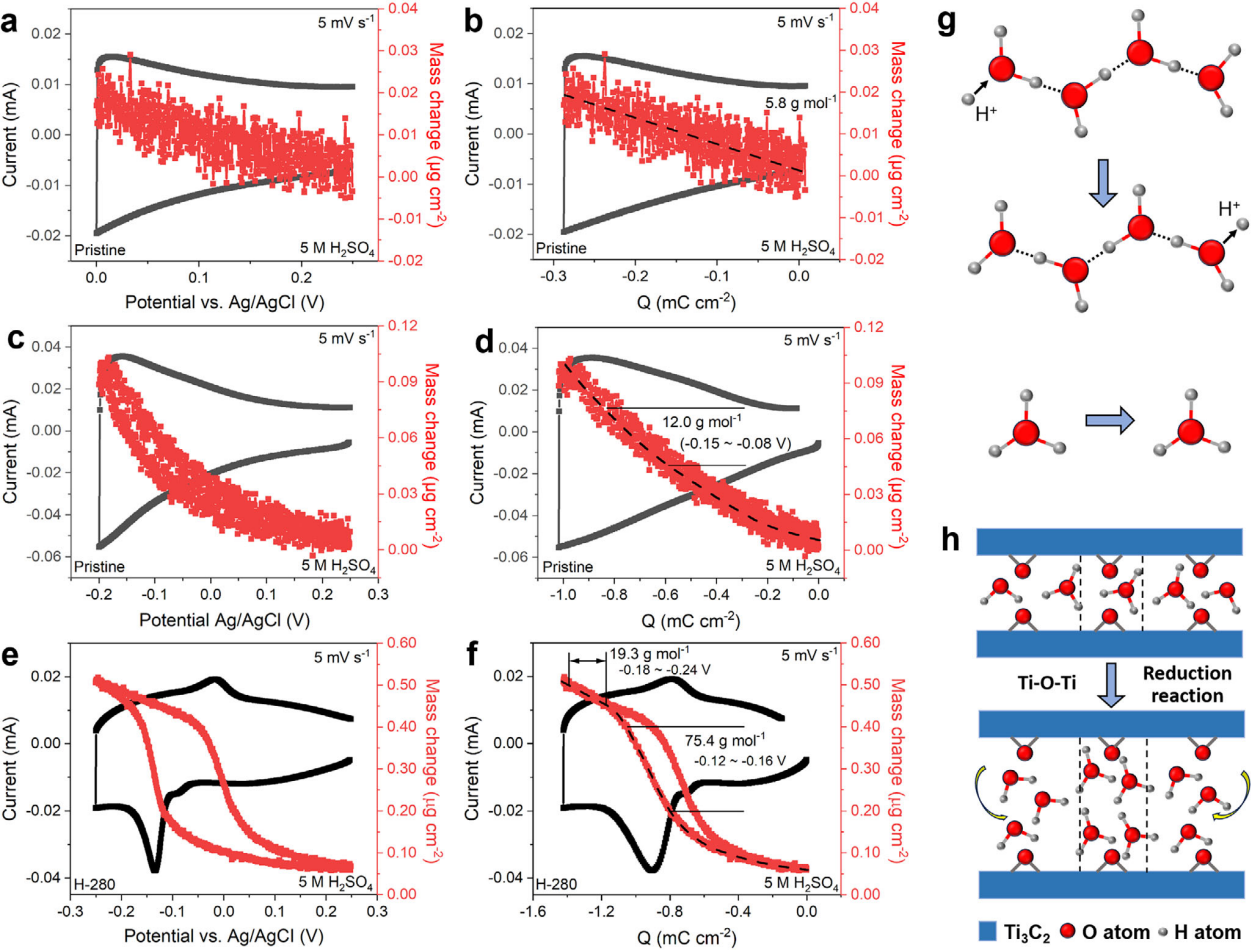
EQCM analysis and actuation mechanism of MXene in 5 M H_2_SO_4_ electrolyte. (a) CV profile and electrode mass response, and (b) electrode mass change versus charge for the pristine Ti_3_C_2_T*_x_* on an Au‐coated quartz crystal substrate, recorded at 5 mV s^−1^ within a potential range of 0 to 0.25 V. EQCM measurements over a broader potential range (−0.25–0.25 V) are presented in (c, e) and (d, f). (g) Schematic illustrations of the Grotthuss mechanism (proton transport via hydrogen‐bond rearrangement along a water chain) and the vehicle mechanism (migration of hydrated proton as a whole). (h) Proposed schematic of interlayer structural evolution in MXene upon reduction of Ti─O─Ti in H_2_SO_4_ electrolyte.

Owing to the hydrophilicity of Ti_3_C_2_T*_x_*, abundant water molecules are adsorbed by terminal groups such as ─OH or ─O─ within the layers. Under van der Waals forces, these water molecules become confined and form a continuous hydrogen‐bond network. Consequently, protons (H^+^) can migrate through the layers via the Grotthuss mechanism (Figure 4g). In contrast, hydrophobic channels formed by halogen terminal groups (e.g., Cl and F) or discontinuous hydroxyl regions favor the transport of hydronium ions through the Vehicle mechanism. Thus, within the initial electric double‐layer region, the predominance of proton hopping over hydronium ion transport results in a low apparent molar mass per transferred charge (5.8 g mol^−1^). As discharge proceeds into the pseudocapacitive region (Figure 4c,d), the average molecular weight per transferred charge gradually increases, reaching approximately 12.0 g mol^−1^ within −0.15–−0.08 V (vs. Ag/AgCl). This trend indicates that during the Ti─O─Ti pseudocapacitive reaction, additional water molecules accompany charge injection compared to the EDLC‐dominated regime.

The EQCM analysis of the high‐capacitance MXene (e.g., H‐280) verifies that electrochemical actuation in acidic electrolytes is governed by reaction‐induced interlayer expansion coupled with water influx. Owing to its higher density of Ti─O─Ti active sites, the H‐280 MXene is intrinsically more hydrophilic. Within the high‐potential region, the average molecular weight per transferred charge remains nearly constant at about 6.7 g mol^−1^—comparable to pristine Ti_3_C_2_T*_x_*—despite the occurrence of Ti═O pseudocapacitive reactions (Figure 4e,f, Figure S10). When the discharge enters the Ti─O─Ti reaction region, the average molecular weight per transferred charge increases abruptly, reaching 75.4 g mol^−1^ within the potential range of −0.16–−0.12 V (vs. Ag/AgCl). Beyond this narrow potential window, however, the value drops sharply to 19.3 g mol^−1^ (corresponding to the molar mass of H_3_O^+^ species), even though the reaction remains within the Ti─O─Ti pseudocapacitive range. This behavior is fully consistent with the trend in interlayer spacing variation. The distinct mass responses observed within the same pseudocapacitive reaction region indicate that the substantial water influx is not directly caused by the insertion of hydronium ions (e.g., H_9_O_4_^+^ or H_11_O_5_^+^). As discussed above, protons rarely exist independently and are stabilized at least as H_3_O^+^ [37]. Moreover, pseudocapacitance proceeds via a Faradaic process, in which chemical adsorption between ions and the electrode is significantly stronger than the physical adsorption in the electric double‐layer formation [39]. Thus, for H‐280, the high density of Ti─O─Ti active sites promotes the simultaneous accumulation of multiple (“multiple” refers to the number of hydrated ions, mainly H_3_O^+^) hydrated ions within confined interlayer regions during reduction (schematically illustrated in Figure 4h). This localized charge accumulation generates sufficient support points to overcome van der Waals interactions between adjacent layers, leading to sudden interlayer expansion and driving a pronounced influx of water molecules from the electrolyte. Once a sufficient number of active sites are engaged to stabilize the expanded 2D framework, subsequent reactions proceed without inducing further significant structural change. The initially reacted sites effectively act as structural anchors that support the expanded configuration. In contrast, when the density of Ti─O─Ti active sites is relatively low, as in the pristine and H‐220 samples, the probability of forming such cooperative interlayer interactions is substantially reduced. Consequently, its maximum achievable interlayer expansion is significantly smaller than that of H‐260 and H‐280 (Figure S11).

In summary, the pseudocapacitive reaction first triggers interlayer expansion, which subsequently accommodates water molecules, rather than directly involving hydronium insertion coupled with structural expansion.

### 2.4 Electrochemical Actuation Performance of MXene Film in Neutral Electrolyte

To further investigate the electrochemical actuation mechanism of MXene‐based actuators, the study was extended to neutral electrolytes. To suppress water electrolysis and expand the electrochemical stability window, a highly concentrated water‑in‑salt (WIS) electrolyte (19.8 M LiCl) was employed, which enables pseudocapacitive behavior within an extended potential range. As shown in Figure 5a,b, when pristine Ti_3_C_2_T*_x_* was used as the working electrode, distinct redox peaks appeared above the open‑circuit potential (OCV) during the first charge–discharge cycle. This observation indicates that a spontaneous pseudocapacitive adsorption process occurs under these conditions (Figure S12), consistent with previous reports by Wang et al. [40]. To identify the functional groups responsible for this spontaneous pseudocapacitive reaction with Li^+^, in situ UV–vis spectroscopy was conducted on pristine Ti_3_C_2_T*_x_* in 19.8 M LiCl electrolyte (Figure S13). Notably, no discernible change in the UV–vis spectrum was observed within this redox potential region, indicating that Ti─O/═O groups are not involved in this process. Furthermore, electrochemical reactions associated with chlorine terminations typically occur at lower potentials (< 0 V, Figure S14). Therefore, the spontaneous pseudocapacitive behavior observed above the OCV is most plausibly attributed to fluorine functional groups.

**FIGURE 5:**
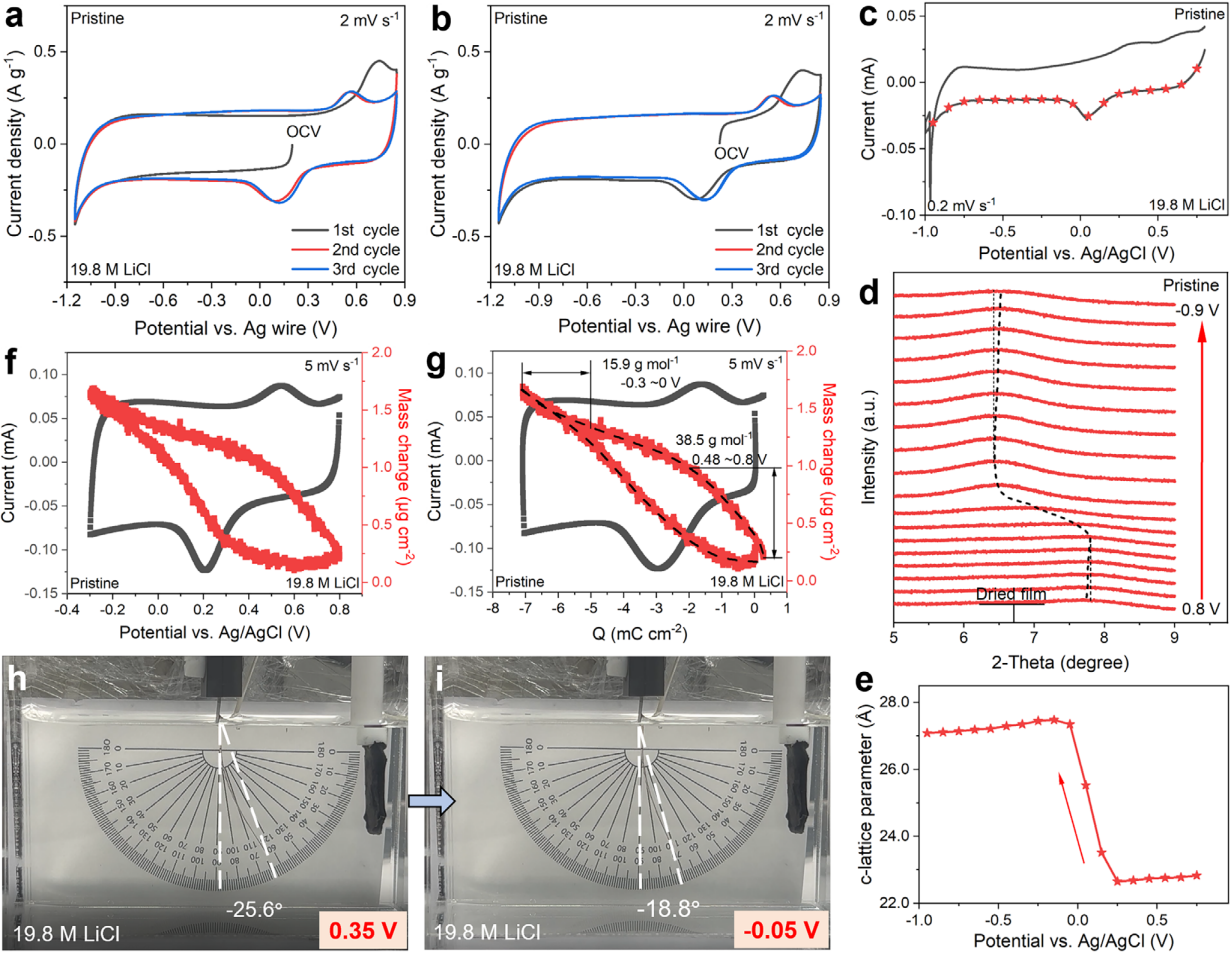
In situ electrochemical characterization and actuation of MXene in 19.8 M LiCl “water‐in‐salt” electrolyte. (a, b) The initial three CV curves of the pristine Ti_3_C_2_T*_x_* electrode at a scan rate of 2 mV s^−1^ within a potential window of −1.15 to 0.85 V. (c) CV curve of the pristine Ti_3_C_2_T*_x_* electrode recorded at a slower scan rate of 0.2 mV s^−1^, along with (d) the corresponding in situ XRD patterns collected during cycling. (e) Evolution of the *c*‐lattice parameter as a function of applied potential. (f) CV profile synchronized with the electrode mass response measured by EQCM, and (g) electrode mass change as a function of accumulated charge for pristine Ti_3_C_2_T*_x_* on an Au‐coated quartz crystal (5 mV s^−1^, −0.3 to 0.8 V). Optical images of the pristine Ti_3_C_2_T*_x_*‐based actuator captured at (h) 0.35 V and (i) −0.05 V (vs. Ag/AgCl) during discharge.

Guided by the established correlation between pseudocapacitive reactions and interlayer spacing variation, in situ XRD was performed to track the structural evolution of MXene interlayers in the neutral electrolyte (Figure 5c–e). As depicted in Figure 5d, extraction of the spontaneously adsorbed ions from the interlayer region results in a concurrent removal of weakly hydrated Li^+^ species and water molecules associated with surface hydroxyl groups. This loss of both structural water and hydrated‐ion support destabilizes the interlayer framework, leading to a rapid collapse of the MXene layers. Consequently, the (002) diffraction peak shifts from ∼6.7° (hydrated state film) to 7.7°, corresponding to a pronounced contraction of the interlayer spacing. Upon subsequent charge reinjection, hydrated Li^+^ ions are rapidly re‐adsorbed at the active sites, restoring the interlayer spacing (Figure 5d,e). This reversible structural breathing behavior is accompanied by an elevated average molecular weight per transferred charge during the redox reaction, as further confirmed by EQCM measurements (Figure 5f,g and Figure S15).

As established, interlayer reduction in MXene reduces film contraction (γ↔γ’ transition, Scheme 1a), which is visually evidenced by electrode curling toward the MXene side at 0.35 V (Figure 5h, Video S3). Mechanistically, such reduction‐driven contraction can generate localized interlayer tearing to relieve stress [41]. Therefore, during the subsequent sharp re‐expansion of the interlayer spacing, a significant portion of the structural expansion is consumed in healing these microcracks rather than contributing to macroscopic bending. This internal structural recovery dissipates the expansion strain, thereby markedly attenuating the macroscopic actuation deformation that would otherwise be expected from interlayer spacing variation (Figure 5i, Figure S16 and S17).

For the H‐280 sample, two distinct pairs of redox peaks appear between −1.15 and 0.85 V (vs. Ag) (Figure S18). The higher‐potential pair, corresponding to the Ti═O groups and proceeding spontaneously (Figures S19 and S20), resembles the behavior of pristine Ti_3_C_2_T*_x_*. Notably, even at the onset of oxygen evolution, the spontaneously adsorbed ions are not fully extracted, thereby limiting their contribution to interlayer spacing variation—consistent with the negligible electrode bending observed within this potential window (Figure S21, Video S4). In contrast, pronounced bending emerges at lower potentials driven by Ti─O─Ti active sites. Although actuation is activated in this region, the overall deformation remains constrained by the hydrogen evolution limit in the neutral electrolyte. This comparison underscores the superior actuation performance of MXene in acidic media, where a more favorable electrochemical window and unique proton–water interactions enable more efficient utilization of redox‐active sites. Ultimately, the dense accumulation of multiple hydrated ions within confined spaces leads to drastically improved actuation, particularly for oxygen‐functionalized MXene.

### 2.5 MXene‐Based ECAs Working in Acidic Solid‐State Electrolyte

The practical feasibility of MXene‐based electrodes for ECAs was demonstrated by fabricating an integrated, ambient‐air‐operable device, in which two MXene strips were sandwiched with PVA‐H_2_SO_4_ gel electrolyte (inset in Figure 6a and Video S5). Upon charging the solid‐state ECA (i.e., operating as a supercapacitor), cations intercalate into the electrode on the side where the potential decreases, thereby expanding the electrode due to increased interlayer spacing. On the opposite side, where the potential increases, spontaneously adsorbed ions are first desorbed (Figure S6). With further polarization potential, anion insertion may occur; however, both processes contribute minimally to electrode deformation. These results demonstrate that voltage‐driven mechanical actuation in this symmetric device architecture is intrinsically feasible, consistent with the bending behavior observed in the optical images (Figure 6b,c).

**FIGURE 6:**
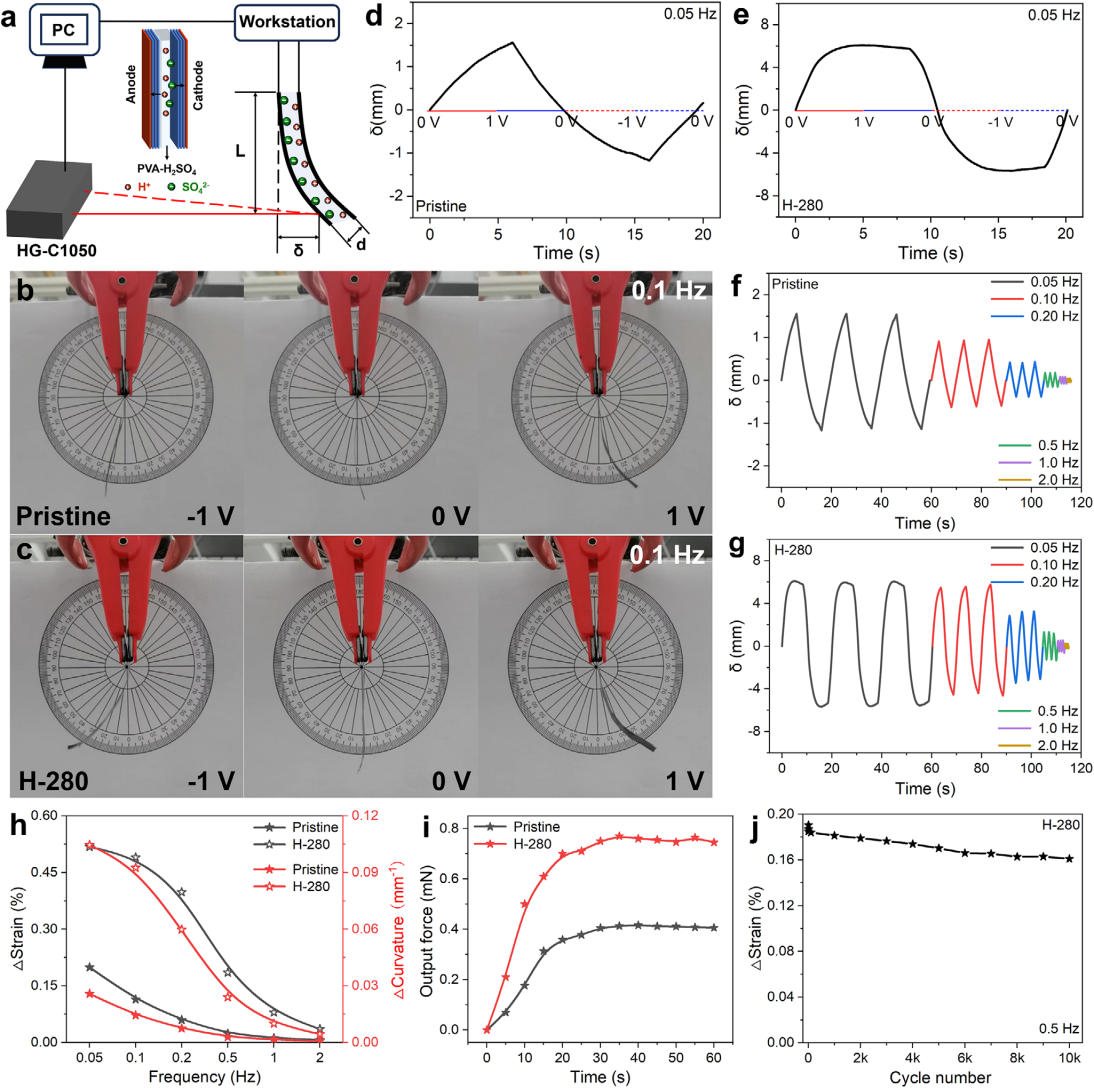
Electrochemical actuation performance of MXene‐based solid‐state actuators. (a) Schematic diagram of the actuator configuration for the MXene|PVA‐H_2_SO_4_|MXene solid‐state ion actuator. Optical image sequences capturing the bending profiles of (b) pristine and (c) H‐280 functionalized actuators driven by ±1 V at 0.1 Hz. Displacement‐time curves of (d) pristine and (e) H‐280 actuators driven by ±1 V at 0.05 Hz. Displacement curves of (f) pristine and (g) H‐280 actuators driven by ±1 V at different frequencies. (h) Strain difference (black symbol) and curvature difference (red symbol) as a function of frequency. (i) Comparison of actuator output force. (j) Cycling stability of the H‐280 actuator, showing strain difference under ±1 V at 0.5 Hz over 10 000 cycles.

The H‐280 ECA exhibits markedly superior performance compared to the pristine device (Figure 6d,e). At 0.05 Hz, it achieves a maximum displacement of 11.77 mm—4.31 times that of the pristine Ti_3_C_2_T*_x_* ECA (2.73 mm). Even at a higher frequency of 0.5 Hz, a displacement of 2.96 mm is maintained, corresponding to an 8.97‐fold enhancement relative to the pristine device (0.33 mm) (Figure 6f,g). These results indicate that the increased density of oxygen functional groups significantly enhances both tip displacement and frequency response of the actuator. Figure 6h illustrates the frequency‐dependent variation of strain and curvature differences for the two solid‐state ECAs. Both parameters decrease consistently with increasing frequency, reflecting the finite response time required for ion intercalation from the PVA‐H_2_SO_4_ gel into the electrode interlayers. At high frequencies, insufficient ion insertion reduces bending efficacy, leading to lower strain and curvature. Benefiting from its enhanced material properties, the H‐280 ECA shows significantly higher performance at 0.05 Hz, with strain and curvature differences of 0.52% (peak‐to‐peak strain of about 1%) and 0.105 mm^−1^, respectively, compared to only 0.20% and 0.026 mm^−1^ for the pristine device. These values are comparable to or surpass those of most previously reported actuators, especially those based on pure MXene electrodes and aqueous electrolytes (Table S1). Furthermore, the H‐280 ECA delivers a high output force, with a steady‐state force approximately twice that of the pristine device (Figure 6i). Nevertheless, this force enhancement is not proportional to the much larger displacement. This discrepancy is likely associated with the pronounced interlayer restructuring and water uptake/release processes in the MXene electrodes (particularly H‐280) during electrochemical cycling, which may dynamically influence the Young's modulus of the film during actuation.

Beyond its excellent electrochemical actuation performance, the H‐280 ECA also demonstrates robust durability, retaining approximately 80% of its initial strain difference after 10 000 cycles at 0.5 Hz under a ±1 V square‐wave voltage (Figure 6j). The gradual decay in strain is likely due to water loss from the PVA–H_2_SO_4_ gel electrolyte and cumulative microstructural degradation of the electrode. Further improvements in cycling stability could be achieved through device‐level optimization, such as advanced encapsulation or tailored electrode–electrolyte interfaces.

## 3 Discussion

Overall, this study elucidates the fundamental mechanisms of electrochemical actuation in MXene‐based ECAs by directly linking tailored surface chemistry and charge‐storage dynamics to macroscopic deformation. Controlled hydrothermal substitution of terminal groups systematically modulates the interlayer environment. Specifically, introducing oxygen functional groups (─O─, ═O) enhances the pseudocapacitive activity of Ti sites while reducing the pristine interlayer spacing, creating a structurally preconditioned state primed for large, stimuli‐responsive dimensional changes. The actuation mechanism is intrinsically coupled to Faradaic processes: The pronounced interlayer expansion in acidic electrolyte does not scale simply with accumulated charge but originates from the dense, localized accumulation of hydrated ions at confined Ti─O─Ti active sites during pseudocapacitive reactions. A solid‐state ECA employing a PVA‐H_2_SO_4_ gel electrolyte demonstrates the practical viability of oxygen‐functionalized MXene (H‐280). Under a ±1 V square‐wave voltage at 0.05 Hz, it achieves a peak‐to‐peak strain difference of ∼1%—a fourfold increase over the pristine Ti_3_C_2_T*_x_* device—while retaining ∼80% strain retention after 10 000 cycles.

This mechanistic framework, connecting atomic‐scale surface chemistry to microstructural evolution and device‐level performance, provides a foundational principle for the rational design of high‐performance, reaction‐driven 2D material actuators. More broadly, the strategy of tailoring electrode–electrolyte interfaces to achieve both high electrochemical actuation performance and robust durability offers a promising avenue for the future development of advanced ECA materials.

## 4 Materials and Methods

### 4.1 Preparation of Ti_3_C_2_T_x_ Colloidal Solution

LiF (1 g) was added to 9 M HCl solution (20 mL), followed by a slow addition of 1 g of Ti_3_AlC_2_ [42] (400 mesh, 11 Technology Co., Ltd., P. R. China) with stirring in an ice bath, and then transferred to an oil bath. After etching for 36 h at 40°C, the product was washed with deionized water by centrifuging until the pH of the supernatant reached 6. The remaining sediment (multilayer Ti_3_C_2_T*_x_*) was mixed with 100 mL deionized water and probe sonicated (400 W) for 1 h under Ar atmosphere and ice bath. Afterward, the mixture was centrifuged for 1 h at 3500 rpm. The resultant dark supernatant was a colloidal solution of single/few‐layer Ti_3_C_2_T*_x_*.

### 4.2 Preparation of Ti_3_C_2_O_y_ Colloidal Solution

KOH (1.5 g) was slowly added to diluted Ti_3_C_2_T*_x_* colloidal solution (35 mL) [24] (2 mg mL^−1^). The suspension was transferred to a 50 mL para polyphenol (PPL) lined stainless steel autoclave purged with Ar at a flow rate of 200 mL min^−1^ for 5 min to remove the air and dissolved oxygen, and heated at 280°C for 20 h (Unless otherwise specified, all samples were hydrothermally treated for 20 h, as no significant differences were observed between samples treated for 20 and 30 h). After cooling down to room temperature, the product was washed with 1 M H_2_SO_4_ and deionized water via centrifugation until the pH of the supernatant reached about 6. The remaining sediment was mixed with 100 mL of deionized water and probe sonicated (power: 400 W) for 20 min under Ar atmosphere and an ice bath to obtain Ti_3_C_2_O*_y_* colloidal solution (denoted as H‐280). Other hydrothermally treated samples at different temperatures were denoted as H‐T (T = hydrothermal temperature in °C).

### 4.3 Preparation of Multi‐Layered Ti_3_C_2_Cl_2_

Ti_3_AlC_2_ [43] (0.3 g) powder was mixed with CdCl_2_ in NaCl/KCl salts (1:3:6:6 weight) using a mortar and pestle. The resulting mixture was heated in an alumina crucible under Ar at 650°C for 5 h with a ramping rate of 5°C min^−1^. After washing with HCl and following DI water, the product was filtrated and dried under vacuum at 110°C for 3 h to obtain Ti_3_C_2_Cl_2_ powder.

### 4.4 Preparation of Vacuum‐Filtered Ti_3_C_2_ Thin Film

Ti_3_C_2_T*_x_* or Ti_3_C_2_O*_y_* colloidal solution was vacuum filtered through a 25 µm thick Celgard 3501 polypropylene membrane. The wet film on the Celgard membrane was collected and dried under vacuum overnight at 80°C, then peeled off to yield flexible, freestanding thin films. Finally, the film was pressed onto a smooth Cu foil under 2 Mpa and peeled off for subsequent electrode fabrication.

### 4.5 Preparation of Ti_3_C_2_ Actuator Electrode in Three‐Electrode System

Freestanding Ti_3_C_2_ thin films (∼1.5 mg cm^−2^) were cut into strips (5 × 35 mm). Subsequently, each MXene strip was firmly adhered to a piece of Kapton tape (60 µm thick) of the same size.

### 4.6 Preparation of PVA‐H_2_SO_4_ Gel Electrolyte

Polyvinyl alcohol (PVA), H_2_SO_4,_ and DI water were mixed in a 1:1:10 mass ratio. Specifically, 1 g PVA was dissolved in 10 mL DI water at 85°C with stirring, cooled to room temperature and then mixed with 1 g H_2_SO_4_ for 30 min to obtain a transparent gel electrolyte [21].

### 4.7 Assembly of Ti_3_C_2_ Film/PVA‐H_2_SO_4_/Ti_3_C_2_ Film Electrochemical Actuators

A Ti_3_C_2_ strip was placed on a glass plate. Then a viscous PVA‐H_2_SO_4_ gel electrolyte was uniformly coated onto the MXene surface. After partial drying to form a solid‐like electrolyte layer, the second strip was carefully aligned and laminated on top. A Celgard 3501 separator was inserted between the two electrodes at the clamping edge to prevent electrical short‐circuiting.

### 4.8 Electrochemical and Actuator Testing

Electrochemical measurements were performed using a CHI660E electrochemical workstation. In the three‐electrode system, YP‐50 activated carbon film and Ag/AgCl electrode (saturated KCl) were used as counter and reference electrodes, respectively. The YP‐50 activated carbon film, with excess capacity, was pressed on the platinum mesh. Cyclic voltammetry (CV) was performed to cycle the actuator electrode, and a digital camera was used to record the corresponding morphological changes. For the solid‐state actuator, a laser displacement sensor (HG‐C1050) was placed on the tip of the actuator to monitor its displacement. The bending angle was then calculated from the measured displacement.

The curvature (*κ*) and strain (*ε*) were calculated using the following formulas:

(1) $$\kappa =\frac{2\delta }{L^{2}}$$

(2) $$\varepsilon =\frac{2d\delta }{L^{2}+\delta^{2}}$$

where *δ* (mm) is the tip displacement of the actuator, *d* (mm) is the thickness of the actuator and *L* is the length of the actuator.

The overall differences in curvature (Δ*κ*) and strain (Δ*ε*) were calculated using the following equations:

(3) $$\Delta \kappa =\kappa_{\max}\;-\kappa_{\min}$$

(4) $$\varepsilon =\varepsilon_{\max}\;-\varepsilon_{\min}$$

where *κ*_max_, *κ*_min_ are the maximum and minimum curvature values, respectively, and *ε*_max_ and *ε*_min_ are the corresponding strain values in an actuation cycle.

### 4.9 In‐Situ XRD Measurements

In situ XRD measurements were carried out on a Bruker D8 diffractometer using a modified three‐electrode cell, with vacuum‐filtered Ti_3_C_2_ film, YP‐50 activated carbon film, and Ag/AgCl (saturated KCl) as the working, counter, and reference electrodes, respectively. The working electrode (Ti_3_C_2_ film, 5 mm in diameter) was attached to a glassy carbon substrate using conductive carbon nanotube adhesive tape (JCHANO Tech Co., Ltd., P. R. China) to prevent curling during charging‐discharging cycling. The YP‐50 activated carbon film was pressed onto the platinum mesh. A Kapton window enabled electrolyte infiltration into the electrode via capillary action while allowing X‐rays to penetrate. XRD patterns were collected during cyclic voltammetry at a scan rate of 0.1 mV s^−1^ with a small potential increment (0.04 V) to ensure sufficient XRD granularity in the 2*θ* range of 5°–9° with a step of 0.01°.

### 4.10 In Situ Electrochemical UV–vis Spectroscopy Measurements

In situ UV–vis measurements were carried out on a Shimadzu UV‐2450 spectrophotometer equipped with an integrating sphere using a modified two‐electrode cell, in which vacuum‐filtered Ti_3_C_2_ film and YP‐50 activated carbon film served as working and counter /reference electrodes, respectively. The Ti_3_C_2_ film (1mg cm^−2^) was mounted on glass, and graphite paper with a central opening was used as the current collector to establish electrical contact with the Ti_3_C_2_ film. The YP‐50 film with high areal loading (∼30 mg cm^−2^) was attached to the graphite paper. Celgard 3501 and 5 M H_2_SO_4_ were used as the separator and electrolyte, respectively. UV–vis spectra were collected during cyclic voltammetry at a scan rate of 0.5 mV s^−1^ with a small potential increment (∼0.027 V) to ensure sufficient UV–vis granularity in the range of 350–900 nm^−1^ with a step of 10 nm s^−1^. Different from the previously reported method [27], spectra signals were collected using the reflection method. For experiments conducted in 19.8 M LiCl electrolyte, an Ag wire reference electrode was employed to facilitate comparison with prior studies [40].

### 4.11 Electrochemical Quartz Crystal Microbalance (EQCM) Measurements

For EQCM sample preparation, diluted Ti_3_C_2_ colloidal solution was drop‐coated on Au‐coated quartz crystals (oscillating frequency *f*_0_ = 5 MHz, Shenzhen RenLux Crystal Co., Ltd). Prior to coating, the quartz crystals were cleaned by oxygen plasma for 5 min to enhance hydrophilicity. After drying overnight at room temperature under a nitrogen flow, the resulting ultrathin (mass loading is about 50 µg cm^−2^) binder‐free Ti_3_C_2_T*_x_* film adhered firmly to the quartz surface and served as the working electrode. The measurement was performed in a three‐electrode system with Ag/AgCl (saturated KCl) as the reference electrode and YP‐50 activated carbon film as the counter electrode. The electrolyte was 5 M H_2_SO_4_, and all EQCM measurements were performed using an eQCM 10M (Gamry) coupled with an electrochemical workstation (Interface 1000, Gamry).

### 4.12 Materials Characterization

X‐ray diffraction (XRD) was carried out on a Bruker D8 advance powder X‐ray diffractometer equipped with copper Kα radiation (λ = 1.54 Å). The cross‐sections and elementary compositions of the Ti_3_C_2_ thin films were characterized by JSM‐IT800 field emission scanning electron microscope (FE‐SEM, JOEL) equipped with an energy‐dispersive X‐ray spectroscope (EDS, Oxford). Atomic‐resolution characterization was conducted on a double spherical aberration corrected scanning transmission electron microscope (STEM, Thermo Fisher Titan Themis G2 60–300) operated at 300 kV. Single‐layer Ti_3_C_2_ samples for TEM observation (FEI Tecnai G2 F30) were prepared by dropping two drops of the diluted Ti_3_C_2_ colloidal solution onto a lacey carbon‐coated copper grid (Beijing XXBR Technology Co., Ltd., P. R. China) and drying under argon flow. Foil samples of vacuum‐filtered Ti_3_C_2_ films for cross‐section STEM observation were prepared using a Zeiss Crossbeam 540 focused ion beam (FIB). Ultraviolet‐visible (UV–vis) spectroscopies were characterized to determine the functional groups using Shimadzu UV‐2450 spectrophotometer.

## Conflicts of Interest

The authors declare no conflicts of interest.

## Supporting information




**Supporting File 1**: advs77097‐sup‐0001‐SuppMat.docx.

**Supporting File 2**: advs77097‐sup‐0002‐VideoS1.mp4.

**Supporting File 3**: advs77097‐sup‐0003‐VideoS2.mp4.

**Supporting File 4**: advs77097‐sup‐0004‐VideoS3.mp4.

**Supporting File 5**: advs77097‐sup‐0005‐VideoS4.mp4.

**Supporting File 6**: advs77097‐sup‐0006‐VideoS5.mp4.

## Data Availability

The data that support the findings of this study are available from the corresponding author upon reasonable request.
