# Supplementary material for: Anomalous Enhancement in MXene‐Based Electrochemical Actuators: Pseudo‐Capacitance‐Induced Multi‐Charge Accumulation in Confined Space
Source: Adv Sci (Weinh). 2026 Aug 14:e77097. Online ahead of print. doi: 10.1002/advs.77097 (PMC13474044; doi:10.1002/advs.77097)
Supplement: Supplementary file 1 [file ADVS-9999-e77097-s003.docx]

**Supplementary Information**

**Anomalous Enhancement in MXene-Based Electrochemical Actuators: Pseudo-Capacitance-Induced Multi-Charge Accumulation in Confined Space**

Jiang Xu^a,*^, Jipeng Feng^a^, Bingqing Hu^b^, Xude Li^b^, Shifei Huang^c*^, Shanhai Ge^a,d*^, Guoxiu Wang^e*^

^a^ College of Electrical, Energy and Power Engineering, Yangzhou University, Yangzhou, Jiangsu 225127, China

^b^ Institute of Intelligent Flexible Mechatronics, Jiangsu University, Zhenjiang, Jiangsu 212013, China

^c^ School of Mechanical Engineering, Yangzhou University, Yangzhou, Jiangsu 225127, China

^d^ Department of Mechanical Engineering, The Pennsylvania State University, University Park, PA 16802, USA

^e^ Centre for Clean Energy Technology, School of Mathematical and Physical Sciences, Faculty of Science, University of Technology Sydney, Sydney, NSW 2007, Australia

* Corresponding author: xuj@yzu.edu.cn (J. Xu); huangsf@yzu.edu.cn (S. Huang); shanhaige@yzu.edu.cn (S. Ge); guoxiu.wang@uts.edu.au (G. Wang)

**Figures:**

**Figure S1** **Schematic illustration of the wave-like morphology.** The wave-like morphology described in this work differs from the ideal two-dimensional film structure. In an ideal 2D film, interlayer variations induce strain solely along the direction normal to the substrate, without causing macroscopic bending of the actuator electrode. However, due to the limited lateral dimensions of the MXene flakes used in this study, the flakes cannot be perfectly aligned parallel to the substrate during vacuum filtration, resulting in partially random stacking orientations. Consequently, interlayer variations generate not only through-plane strain but also in-plane (tangential) strain. As shown in the figure, the resulting cross--sectional HAADF-STEM morphology exhibits a wave-like appearance, and we therefore define this structure as a wave-like morphology.

For the vacuum-filtered H-280 film, removal of interlayer water upon drying induces significant contraction. Owing to the random stacking orientation of the flakes, the resulting in-plane strain is accommodated through localized interlayer separation rather than macroscopic deformation, allowing the film to retain its overall structural integrity.

**Figure S2** Synthesis scheme of Ti_3_C_2_T*_x_* and Ti_3_C_2_O*_y_*.

**Figure S3** EDS element analysis of pristine T_3_C_2_T*_x_* and hydrothermally treated samples under different temperatures and treatment times. (a) C/Ti ratio, (b) F/Ti ratio, (c) Cl/Ti ratio, and (d) O/Ti ratio.

**Figure S4** High-resolution O 1s and Ti 2p spectra of the pristine Ti_3_C_2_T*_x_* and H-280 films.

**Figure S5** Optical images of (a) pristine Ti_3_C_2_T*_x_* and (b) H-280 actuator electrodes measured at a scan rate of 5 mV s^-1^ in 5 M H_2_SO_4_ electrolyte.

**Figure S6** (**a**) Initial three CV curves of the H-280 electrode at a scan rate of 10 mV s^-1^ within a potential window of -0.5 to 0.35 V in 5 M H_2_SO_4_ electrolyte. Partial Ti=O active sites underwent pseudocapacitive reactions with hydrogen ions during the immersion stage. (**b**) Schematic illustration of the charging process in an H-280|PVA-H_2_SO_4_|H-280 actuator. The cathode exists two distinct ion-transport stages: initial cation extraction followed by anion adsorption. In contrast, the anode undergoes continuous cation adsorption throughout the charging process.

**Figure S7** (**a**) CV curves of pristine Ti_3_C_2_T*_x_*, H-220, H-260, and H-280 film electrodes at a scan rate of 5 mV s^-1^. (**b**) Gravimetric rate performance of pristine Ti_3_C_2_T*_x_*, H-220, H-260, and H-280 film electrodes calculated from cyclic voltammograms at different scan rates.

**Figure S8** (**a**) Schematic illustration of the cell component composition used for *in-situ* UV-Vis measurements. (**b**) Optical image of the cell for *in-situ* UV-Vis measurement in reflectance mode.

**Figure S9** CV curves of (a) pristine Ti_3_C_2_T*_x_* and (b) H-280 recorded during *in-situ* electrochemical UV-Vis spectroscopy (Scan rate: 0.5 mV s^-1^; reference electrode: YP-50 activated carbon) and *in-situ* XRD (Scan rate: 0.1 mV s^-1^; reference electrode: Ag/AgCl) measurements. The potential difference between YP-50 activated carbon and Ag/AgCl is about 0.45 V.

**Figure S10** (a) CV profile and electrode mass response and (b) electrode mass change versus charge for H-280 on an Au-coated quartz crystal substrate recorded at 5 mV s^-1^ within a potential range of 0.05 to 0.25 V in 5 M H_2_SO_4_ electrolyte.

**Figure S11** Schematic diagram illustrating interlayer changes in a) pristine Ti_3_C_2_T*_x_* / H-220 and b) H-260 / H-280, before and after the reduction reaction of Ti–O–Ti bonds.

The exceptionally high dehydration energy of H_3_O^+^ (11.66 eV for H_3_O^+^ → H^+^ + H_2_O) prevents its deprotonation. Consequently, during Ti-O-Ti reduction in acidic electrolyte, H_3_O^+^ ions remain tightly adsorbed in their hydrated form within the confined interlayer space (dashed region). This adsorption directly accounts for the observed sudden increase in interlayer spacing.

Compared to H-260/H-280, the pristine/H-220 sample exhibits a larger initial interlayer spacing due to abundant –F, –Cl, and –OH groups. However, these terminal groups hinder the simultaneous formation of Ti–O–Ti on both top and bottom layers, limiting the amount of hydronium ions (H₃O⁺) that can accumulate in the confined interlayer region. Consequently, the interlayer expansion during reduction is much smaller in pristine/H-220 than in H-260/H-280.

**Figure S12** CV of the pristine Ti_3_C_2_T*_x_* film electrode in 19.8 M LiCl electrolyte at a scan rate of 2 mV s⁻¹ over different potential windows: (a) –1.15 to 0 V, (b) 0 to 0.85 V, and (c) –1.15 to 0.85 V.

**Figure S13** *In-situ* electrochemical UV-Vis spectroscopy of the pristine Ti_3_C_2_T*_x_* film electrode in 19.8 M LiCl. (a) CV curves *vs*. Ag wire with a scan rate of 2 mV s^-1^; UV-Vis reflectance spectra measured at different potentials during (b) discharge (red arrow) and (c) charge (blue arrow).

**Figure S14** CV measurements of the Ti_3_C_2_Cl_2_ electrode in 19.8 M LiCl electrolyte at a scan rate of 10 mV s⁻¹ over the potential windows of (a) -0.8 to 0.85 V and (b) 0 to 0.85 V.

**Figure S15** EQCM measurement of pristine Ti_3_C_2_T*_x_* on an Au-coated quartz crystal in a narrower potential range (-0.3 to 0.3 V) to exclude redox contributions at higher potentials. (**a**) CV profile synchronized with electrode mass response; (**b**) electrode mass change versus charge recorded at 5 mV s^-1^.

**Figure S16** Optical images of the pristine Ti_3_C_2_T*_x_*-based actuator electrode recorded at (**a**) 0.35 V and (**b**) 0.75 V (*vs.* Ag/AgCl) during charge. Measurements were conducted in a three-electrode cell using Ag/AgCl as the reference electrode and activated carbon as the counter electrode.

**Figure S17** CV curves and corresponding bending angles of the pristine Ti_3_C_2_T*_x_*-based actuator electrode measured at 10 mV s^-1^ in 19.8 M LiCl electrolyte.

**Figure S18** CV measurements of the H-280 film electrode at a scan rate of 2 mV s⁻¹ in 19.8 M LiCl electrolyte over the potential windows of (a) -1.15 to 0 V, (b) 0 to 0.85 V, and (c) -1.15 to 0.85 V.

**Figure S19** (**a**) The initial three CV curves of the pristine Ti_3_C_2_T*_x_* electrode at a scan rate of 2 mV s^-1^ within a potential window of -1.15 to 0.85 V. (**b**) UV-Vis reflectance spectra of H-280 films: as-prepared, and after 48h soaking in 0.1 M and 19.9 M LiCl.

**Figure S20** *In-situ* electrochemical UV-Vis spectroscopy of the H-280 film electrode: (a) CV curves of H-280 *vs*. Ag wire measured in 19.8 M LiCl at a scan rate of 2 mV s^-1^; UV-Vis reflectance spectra of H-280 measured at different potentials during (b) discharge (red arrow) and (c) charge (blue arrow).

**Figure S21** H-280 actuator measured in 19.8 M LiCl electrolyte using a three-electrode system: (a) CV curve of the H-280 actuator electrode; (b) bending angle of the H-280 actuator electrode measured at different potentials during discharge and charge; optical images of the H-280 actuator electrode taken during discharge at (c) -0.75 V and (d) -1.15 V; optical images taken during charge at (c) -1.05 V and (d) -0.65 V.

**Tables:**

**Table S1.** Representative properties of MXene-based ECAs from recent literature.

| Electrode materials^a^ | Electrolyte^a^ | Voltage window  (V) | Frequency  (Hz) | Strain  (%) | Stability  (%) | Ref. |
| --- | --- | --- | --- | --- | --- | --- |
| Ti_3_C_2_T*_x_* | H_2_SO_4_/PVA | 1.2 | 0.001 | 0.26 | 80.4 (10,000)^b^ | [1] |
| Ti_3_C_2_T*_x_*  /MC | TPU/CMP with LiBr | 2 | 0.001 | 0.541 | 79.8 (5,000) | [2] |
| TBA-Ti_3_C_2_T*_x_* | TPU/CMP with LiBr | 1.7 | 0.001 | 0.771 | 102.6 (10,000) | [3] |
| Ti_3_C_2_T*_x_*  /CNT | TPU with EMIBF_4_ | 5 | 0.1 | 1.54 | ~83 (10,000) | [4] |
| MXene/PEDOT:  PSS/AgNW | Nafion with EMImBF_4_ | 1 | 0.1 | 0.48 | 91.9 (10,000) | [5] |
| Ti_3_C_2_T*_x_*-PEDOT:PSS | Nafion with EMImBF_4_ | 2 | 0.1 | 1.37 | 97  (18,000) | [6] |
| Ti_3_C_2_T*_x_*/CNF  /PPy | Nafion with EMImBF_4_ | 1 | 0.1 | 1.6 | 91.03 (18,000) | [7] |
| Ti_3_C_2_T*_x_*/CNF  /EGaIn | MAF/EMIMBF_4_/PVDF | 6 | 0.1 | 1.6 | 91.2 (10,000) | [8] |
| Ti_3_C_2_T*_x_* | H_2_SO_4_/PVA | 2 | 0.05 | 0.4 | N/A | **This work** |
| H-280 | H_2_SO_4_/PVA | 2 | 0.05 | 1 | 80 (10,000) | **This work** |

^a^ PVA = polyvinyl alcohol; PEDOT:PSS = poly(3,4-ethylenedioxythiophene):poly(styrenesulfonate); EMImBF_4_ = 1-ethyl-3-methylimidazoliumtetrafluoroborate; PPy=polypyrrole; PVDF = polyvinylidene fluoride; MC = methylcellulose; CNF = cellulose nanofibers; EGaIn = eutectic gallium-indium.

^b^ Cycling number

**Videos:**

**Video S1** Deformation of the pristine Ti_3_C_2_T*_x_* electrode during cyclic voltammetry at 5 mV s^-1^ in 5 M H_2_SO_4_ (0.3 V→-0.5 V→0.3V). (10× faster speed)

**Video S2** Deformation of the H-280-based electrode during cyclic voltammetry at 5 mV s^-1^ in 5 M H_2_SO_4_ (0.3 V→-0.5 V→0.3V). (10× faster speed)

**Video S3** Deformation of the pristine Ti_3_C_2_T*_x_* electrode during cyclic voltammetry at 10 mV s^-1^ in 19.8 M LiCl electrolyte. (0.85 V→-1.15 V→0.85V). (10× faster speed)

**Video S4** Deformation of the H-280 electrode during cyclic voltammetry at 10 mV s^-1^ in 19.8 M LiCl electrolyte. (0.85 V→-1.15 V→0.85V). (10× faster speed)

**Video S5** Motion of the two-strip solid-state ECA soft tweezer during opening and closing at a frequency of 0.05 Hz under a ±1 V square-wave voltage. (5× faster speed)

**References**

[1] D. Pang, M. Alhabeb, X. Mu, Y. Dall’Agnese, Y. Gogotsi, Y. Gao. Electrochemical Actuators Based on Two-Dimensional Ti_3_C_2_T*_x_* (MXene). *Nano Lett.* **2019**, 19, 7443–7448. <https://doi.org/10.1021/acs.nanolett.9b03147>.
[2] S. Chen, J.-H. Ciou, F. Yu, J. Chen, J. Lv, P. S. Lee. Molecular-Level Methylcellulose/MXene Hybrids with Greatly Enhanced Electrochemical Actuation. *Adv. Mater.* **2022**, 34, 2200660. <https://doi.org/10.1002/adma.202200660>.
[3] S. Chen, S. F. Tan, H. Singh, L. Liu, M. Etienne, P. S. Lee. Functionalized MXene Films with Substantially Improved Low-Voltage Actuation. *Adv. Mater.* **2024**, 36, 2307045. <https://doi.org/10.1002/adma.202307045>.
[4] W. Zhang, K. Jin, Z. Ren, L. Li, L. Chang, C. Zhang, R. Wang, B. Li, G. Wu, Y. Hu. High-Performance MXene/Carbon Nanotube Electrochemical Actuators for Biomimetic Soft Robotic Applications. *Adv. Funct. Mater.* **2024**, 34, 2408496. <https://doi.org/10.1002/adfm.202408496>.
[5] L. Liu, C. Wang, Z. Wu, Y. Xing. Ultralow-Voltage-Drivable Artificial Muscles Based on a 3D Structure MXene-PEDOT:PSS/AgNWs Electrode. *ACS Appl. Mater. Interfaces* **2022**, 14, 18150. <https://doi.org/10.1021/acsami.2c00760>.
[6] S. Umrao, R. Tabassian, J. Kim, V. H. Nguyen, Q. Zhou, S. Nam, I.-K. Oh. MXene artificial muscles based on ionically cross-linked Ti_3_C_2_T*_x_* electrode for kinetic soft robotics *Sci. Rob.* **2019**, 4, aaw7797. <https://doi.org/10.1126/scirobotics.aaw7797>.
[7] B. Wang, P. Huang, B. Li, Z. Wu, Y. Xing, L. Liu. Construction of MXene interlayer conductive ion transport channel by polypyrrole-coated cellulose nanofibers for ultralow voltage high-performance ionic soft actuators. *Sensor. Actuat. B-Chem.* **2025**, 422, 136649. <https://dor.org/10.1016/j.snb.2024.136649>.
[8] L. Chen, L. Zhang, T. Wu, H. Song. High-performance ionic actuator reinforced by three-dimensional electrode of MXene/cellulose nanofibers/eutectic gallium-indium trilayer structure. *Mater. Today Commun.* **2025**, 46, 112807. <https://doi.org/10.1016/j.mtcomm.2025.112807>.
